# Can colorectal cancer mass-screening organization be evidence-based? Lessons from failures: The experimental and pilot phases of the Lazio program

**DOI:** 10.1186/1471-2458-8-318

**Published:** 2008-09-19

**Authors:** Antonio Federici, Alessandra Barca, Diego Baiocchi, Francesco Quadrino, Sabrina Valle, Piero Borgia, Gabriella Guasticchi, Paolo Giorgi Rossi, The Colon-Rectal Cancer Screening Working Group

**Affiliations:** 1 Agency for Public Health, Lazio Region, via di S. Costanza 53, 00198, Rome, Italy; 2 Italian Ministry of Health, Centre for Disease Prevention and Control, Rome, Italy

## Abstract

**Background:**

Screening programmes should be organized to translate theoretical efficacy into effectiveness. An evidence-based organizational model of colorectal cancer screening (CRCS) should assure feasibility and high compliance.

**Methods:**

A multidisciplinary Working Group (WG), reviewed literature and guidelines to define evidence-based recommendations. The WG identified the need for further local studies: physicians' CRCS attitudes, the effect of test type and provider on compliance, and individual reasons for non-compliance. A survey of digestive endoscopy services was conducted. A feasibility study on a target population of 300.000 has begun.

**Results:**

Based on the results of population trials and on literature review the screening strategy adopted was Faecal Occult Blood Test (FOBT) every two years for 50–74 year olds and, for positives, colonoscopy. The immunochemical test was chosen because it has 20% higher compliance than the Guaiac. GPs were chosen as the preferred provider also for higher compliance. Since we observed that distance is the major determinant of non-compliance, we choose GPs because they are the closest providers, both geographically and emotionally, to the public.

The feasibility study showed several barriers: GP participation was low, there were administrative problems to involve GPs; opportunistic testing by the GPs; difficulties in access to Gastroenterology centres; difficulties in gathering colonoscopy results; little time given to screening activity by the gastroenterology centre.

**Conclusion:**

The feasibility study highlighted several limits of the model. Most of the barriers that emerged were consequences of organisational choices not supported by evidence. The principal limit was a lack of accountability by the participating centres.

## Background

Colorectal cancer screening (CRCS) for average-risk individuals using the faecal occult blood test (FOBT) is effective in reducing mortality from CRC and its use is generally recommended [1-4].

The challenges of disseminating evidence-based interventions are shared by stakeholders, researchers, the intended providers, the intended consumers [5] and, of course, governmental health agencies.

While quality-assurance monitoring is carried out to ensure safe procedures and practice in every clinical aspect of a screening-programme [6], planning and organisation are the only aspects not routinely monitored, yet are the strongest tools to assure the effectiveness of screening [7].

Well-planned interventions have been associated with statistically greater results, particularly for CRCS [8]. Unfortunately, the organizational aspects of an intervention are not usually subject to the same evidence-oriented criteria we apply to medical technologies or clinical procedures.

The impact of CRCS is directly related to its ability to involve the target population, to detect cancers and high-grade adenomas, and to assure patient safety; therefore the main aspects to address at the local level when planning and implementing a CRC screening program are the following:

- How to obtain high compliance [8,9].

- Which Type of screening test among those available (colonoscopy, flexosygmoidoscopy, FOBT Guaiac and the Immunochemical) [10-12].

- Screening knowledge and attitudes of the involved Physicians (GPs and gastroenterologists). [13,14].

- The GP's role in the screening programme. [1,15,16].

- The available Endoscopy resources and the additional workload in case of screening. [3,4].

This paper describes the research involved in planning a colorectal cancer screening program in the Lazio region, Italy, and reports the results of the feasibility study conducted to test the organisational model.

## Methods

### Setting

The Lazio region has 5.3 million inhabitants and includes the city of Rome; the CRCS target population (i.e. people aged 50–74) is 1.5 million.

The Regional Public Health Agency (RPHA) is a technical branch of the regional government of Lazio: it coordinates, monitors and evaluates breast and cervical cancer screening in the 12 Local Health Units. The local government gave a mandate to the RPHA to organize CRCS, with an experimental phase, and a feasibility study [17-22].

### The Colorectal Cancer Working Group (CRCWG)

The method adopted was based on organizing a Colorectal Cancer Working Group (CRCWG) made up of the principle professional figures involved: experts in gastroenterology, epidemiology, screening program organizers, general practitioners; representatives of the physicians' unions and of the regional scientific societies of endoscopists and gastroenterologists. The CRCWG reviewed the guidelines available, giving greater weight to those from governmental bodies; it also reviewed the literature regarding the characteristics of FOBT. They made operational recommendations based on those found in the literature; identified additional cognitive needs in cases where the evidence in the literature was insufficient or inapplicable; for these they identified general and specific objectives.

### The experimental phase

For each objective identified an experimental study or specific intervention was planned and carried out according to the study design or methods listed in table 1. The methods from already published studies are not described further.

**Table 1:** Main studies/interventions conducted and main results achieved during the experimental phase conducted by the Lazio Regional Public Health Authority.

**Objectives**	**Study/intervention ** **conducted**	**Main results**
To describe GPs' the knowledge, attitudes and recommendations about colorectal cancer screening.	Survey of the GPs [18]	24% of the GPs correctly recommended screening for CRC; 22% did not recommend any; 6% under-recommended and 47% over-recommended. 22% of GPs recommended inappropriate follow up tests for patients with positive FOBT.

To describe attitudes and recommendations about colorectal cancer screening of the endoscopy centre physicians.	Survey of the endoscopy center's physicians [19]	Colonoscopy was perceived as the most effective screening test and was the most recommended (80%). FOBT was recommended by 61% of physicians and flexosigmoidoscopy by 11%. 50% over-recommended screening.

To evaluate the effect of the provider (GPs versus hospital) on compliance FOBT screening.	Randomised controlled trial [20]	24.5% of 1192 GPs agreed to participate in the trial. The compliance with the GP was 54% vs 17% with the hospital (RR 3.4; 95%IC 3.1–3.7). There was a high variability in the compliance obtained by the GPs. GPs with more than 25 patient visits per day and those who incorrectly recommended screening had lower compliance (OR 0.74, IC95% 0.57–0.95 and OR 0.76, IC95% 0.59–0.97, respectively).

To assess the effect of the type of FOBT, Guaiac or immunochemical, on compliance.	Cluster-randomised trial [21].	The immunochemical test (OC-Hemodia, Eiken) had a compliance of 35.8% and the Guaiac of 30.4% (RR 1.20; CI95% 1.02–1.44). The Guaiac test had a higher prevalence of positives (10.3% vs 6.3%); and had higher variability in the results.

To identify determinants of non-compliance to FOBT screening.	Case-control study nested in the trial [22].	About 31% of non-compliant people reported never receiving the letter offering free screening; 17% of the sampled population had already been screened. The major reason for non-compliance was "lack of time" (30%), the major determinant of compliance was the distance from the test provider: OR > 30 minutes vs < 15 minutes 0.3 (95%CI = 0.2–0.7).

To define criteria for a quality assurance program for CRC screening endoscopy.	A multidisciplinary panel consensus	A system of quality indicators was created: protection of "users" rights; location in which endoscopy is performed; medical and non-medical staff skills and training in colonoscopy and screening procedures; availability of CRCS-specific management protocols; technical and professional processes; early outcomes evaluation; adverse effects and follow-up management.

To estimate increase in colonoscopies resulting from screening.	Analysis of administrative databases.	Assuming a FOBT positivity rate of 3.5%, a 50% compliance rate, we estimated that nearly 50% more colonoscopies would be required.

The study was submitted and approved by the Committee for Ethics in Screening of the Regional Agency for Public Health, 16^th ^June 2002, approval n°1.

### The endoscopy resources

We analysed the administrative database of hospital records and the distribution of CCR admissions in the previous three years. According to the results of the analysis we selected the endoscopy centres with the highest volume (more than 500 hospitalizations for CRC or adenomas) and at least one centre in each province, to make second level testing feasible in rural areas.

The CRCWG reviewed the literature regarding quality of endoscopic services and defined quality indicators from a disease management perspective, and addressed particular aspects of the relationship between health professionals and patient safety. The endoscopic centres selected were evaluated based on these criteria.

To estimate the number of colonoscopies that would result from extending CRCS to the whole region we conducted a survey of the yearly census of the digestive endoscopy centres by the Regina Elena Institute of Rome and the Italian Society of Digestive Endoscopy (SIED) [19]. We estimated the amount of additional work that would result from screening, according to the following assumptions: a) the demand for colonoscopies produced by the screening program to be entirely independent of current demand for endoscopic exams, as they involve asymptomatic subjects; b) the screening exams were to be performed by facilities located in the subject's area of residence; c) a participation rate of 50% to the hypothetical campaign, and 3,5% of FOBT positivity.

### The information system

To develop the organizational model we referred to RPHA strategic choices (disease management approach, according to a clinical governance framework [16,23]) and to our original data.

According to the clinical governance framework, to assure citizens/patients safety we also planned a clinical risk management programme, using a systemic preventive approach and applying the HFMEA protocol [24]. The results have been incorporated into the organisational model and the management software.

### The feasibility study

A feasibility study on a target population of 300,000 has been started according to the results of the decision-making process. Here we present the results of the first two years of activity, i.e. the first round. The following process indicators are reported to summarise the results of the feasibility study: the number of centres that started the program; the proportion of GPs that agreed to participate in the program; the proportion of the target population that have been contacted (invited); the proportion of people returning the test among the invited population; the positivity rate; the compliance to colonoscopy; the waiting time for colonoscopy.

All 20 gastroenterology centres included in the programme were visited by the RPHA before the program began. The centres that actually started to screen were visited a second time to assess and evaluate the program's progress and to identify critical points.

The screening strategies adopted were consistent with the Ministry of Health guidelines on CRCS, consequently the feasibility study did not need to be submitted to the Committee for Ethics.

## Results

### The evidence collection and production

According to the main evidence, the CRCWG adopted as the screening strategy: FOB testing every two years for 50–74 year olds and, for positives, colonoscopy. We chose FOBT (OC-Hemodia, Eiken) based on the results of population trials [2], and the better compliance to FOBT than flexible sigmoidoscopy in Lazio [25]. All the high-volume endoscopy centres (more than 500 colonoscopies per year), and at least one in each province, were involved to face the predicted 50% increase in the colonoscopy burden induced by screening.

The critical needs identified are listed in table 1; objectives and study or intervention design are also shown. In order to obtain the necessary information to organize the most effective model of mass screening, according to CCRWG indications, we studied the following topics: physicians' attitudes and practices, type of FOBT, test provider, and individual reasons for non-compliance [17].

The main results of the studies/interventions carried out by the RPHA during the experimental phase are shown in table 1; most have already been published [17-22].

The RPHA utilized all the information collected by the above-mentioned studies to determine the best organizational model as summarized in table 2.

**Table 2:** Use of data gathered to define the organizational model.

**Question**	**Experimental evidence/elements from**	**Organizational choices**
Which Organizational framework?	▪ Literature on organisation topics ▪ RPHA Mission	Disease management as a factor to achieve effectiveness

Which Screening test?	▪ Literature evidence ▪ European Commission Recommendation [4] ▪ FOBT has a compliance of 3.1 compared to FS [25]	FOBT as screening test

Which FOBT type?	▪ Literature evidence ▪ The immunochemical test had a 20% higher compliance than Guaiac. ▪ Higher variability in the results obtained with the Guaiac test than the immunochemical [21].	Immunochemical test as FOBT test

Which FOBT provider?	▪ The compliance to the FOBT with GPs was 3.4 times higher than compliance with the hospital, independent of the type of test and geographical area [20]	General Practitioners as main provider

What the GPs think about screening? What they can do?	▪ GPs currently do not correctly follow up a positive FOBT [18]. ▪ There was high variability among GPs: GPs with a heavy work load and those who incorrectly recommended FOBT for CRCS obtained lower compliance [20]	the GPs are not required to participate in the program, but they receive an economic incentive if they do.

Reference diagnostic Centre	▪ Number of CRC cases treated by hospital in the previous 3 yrs plus a guarantee of accessibility in rural areas ▪ A shared system of quality indicators and standards ▪ Attending a re-training course	▪ Centres belonging to Hospitals that have > 500 patients ▪ Centres complying with quality indicators

Why people do not respond to screening invitation?	▪ The first reported reason for non-compliance was "lack of time" (30%). ▪ The major determinant of compliance was the distance from the test provider. [22]	Territorial zoning regardless of administrative borders

Who manage the follow up of positives?	▪ GPs undependable for correct follow-up [18] ▪ Gastroenterologists not skilled in managing mass screening criteria [19] ▪ Neither physicians at endoscopy centers nor GPs tend to follow screening guidelines.	▪ Centralized management ▪ Guidelines-based software

Which software for screening management?	▪ Previous breast cancer screening experience about non-efficiency of in-house softwares, different for each Local Health Unit ▪ Mission: disease-management oriented	▪ Web based software

For each question, the experimental evidence or the elements influencing the final choice are reported

### The organisational model (figure 1)

**Figure 1:**
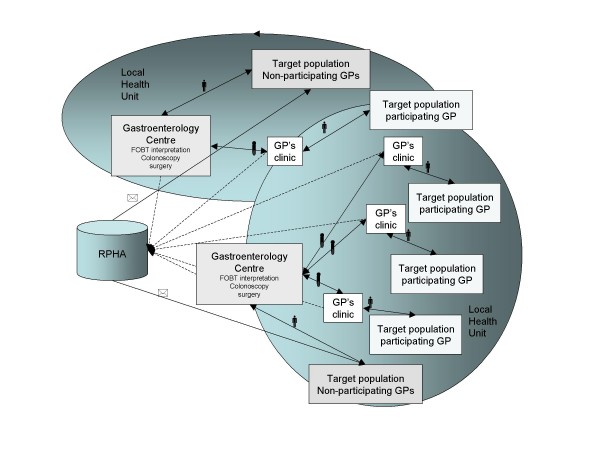
**The dotted lines represents the information flow, the solid lines represents the material flows.** The test tubes represents the faecal samples transported from the GPs' clinics to the gastroenterology centre; the men represent the target population going to the test provider to pick up and return the samples; the letter envelopes represent the invitation by the program to the target population. In the region there are 12 Local Health Units and 20 gastroenterology centres, the target population of each GP and gastroenterology centre is not necessarily restricted to the LHU borders.

From the evidence found, we defined an organizational model, an information system, and software.

The principal characteristics of the organizational model are:

1. to use a disease management approach, including patient treatment and follow-up, and managed according to the principles of clinical governance.

2. the GP is the preferred provider because of its proximity to the healthy population.

3. the GPs are not required to participate in the program, but they receive an economic incentive if they participate: about 10 € per person screened, based on an agreement between the Regional Health Authority and the GPs' trade unions.

4. to offer FOBT as the first-level test (immunochemical method based on latex agglutination with automated analytical procedure). The patients of participating physicians are invited to pick up the test at the GP's office; all patients of non-participating doctors are directed by the local health unit to pick up the test at the local gastroenterology/endoscopy center; the letters were mailed by the Regional Agency for Public Health using centre-specific templates (timetable, addresses and letterhead), but the timing was determined by the gastroenterology centre according to their availability for providing tests and colonoscopies; A reminder was sent to non-responders after three months. The GPs decided when and how (phone, letter or other) to contact their target populations.

5. the result of the test was automatically sent by mail if negative; if positive, the result should have been communicated by phone and letter, in the same phone contact a date for colonoscopy should have been fixed.

6. the gastroenterology centres (20 in the region) collect all the faecal samples and perform the immunochemical analysis and plan the colonoscopy for all the positive samples (and possibly immediate polipectomy).

7. the target population refers to the closest gastroenterology centre, independent of administrative borders

8. the Local Health Unit must establish the administrative framework, and serve as the program's liaison with the GPs and the gastroenterology centres.

### The Information System

A fundamental part of the organisational model is the information system. It is directly managed by the RPHA. The IS is structured in three sections: because there is no cancer registry in our region, the information about incidence and mortality is taken from the integration of the mortality database, the hospital discharge registry; the information about the screening process and outcomes is reported by the on-line record of each screening episode; an adverse events and near-misses information system has been planned.

A fundamental part of the information system as a tool in disease management is the software. We developed a web-based application with the following characteristics: instant access to information by any user, no need for system updates, few hardware needs by the user, information centralized in a single database, possibility of external housing for the server platform, integration with other technological tools (i.e. immuno-test analysers) and with databases (hospital discharge records, out-patient records, mortality database); automatic quality controls to assure that the health professionals involved comply with protocols.

### Results of the feasibility study

The results of the feasibility study are summarised in table 3. The results of the feasibility study can be summarised as follows (table 3, last column): half of the centres started the program, only one invited the entire target population in the two-year round, GP participation was low but varied greatly; the compliance to FOBT with the gastroenterology centre was 16.6%. On the other hand, compliance rates observed for the GPs were not comparable, since we could not determine the denominator of the ratio (i.e. the invited population); in fact most of the GPs suggested the test to patients who came to their office for other reasons, and did not contact the entire target population.

**Table 3:** Results of the feasibility study: process indicators and critical points

**Indicators**	**values**	**range among ** **centres**	**critical points**
**Program activation**			
target population	300000		
N of centres started	50% (10/20)		See discussion
population of active centres	65922 (22,0%)		

*the following data refer only to the active centres*			

**GPs participation**			
% of GP that agreed to participate	10,8% (57/526) 60,0% (57/95)$	(0,0 – 90,0)	GPs were actually involved in only 6 centres: in some LHUs, administrative barriers prevented the participation; no interest by the GPs; some GPs initially agreed but never send the list of target population.
**Program extension (invited population/target)**			
Total	41,1% (65922)		Most of the GPs do not actively invite, but only distribute the tests opportunistically: difficult to exactly determine the contacted population.
GP	21,0% (20315)	(42,2–8,0)	
Hospital	50,1% (45607)	(100,0–14,4)	Too slow invitation rhythm: the program did not cover the whole target population within the two-year period.
**Compliance to FOBT (proportion of returned test/invited population)**			
all	26,5% (27124)		
GP	79,9% (4258)	(73,1 – 91,8)	Impossible to monitor what the GP does. The compliance is biased because of opportunistic strategies, without active invitation by the GP.
Hospital	16,6% (22866)	(4,7 – 34,1)	Low compliance: no accessibility
**Positivity rate**			
	7,1% (6908)	(3,2 – 9,2)	For 288 (%) tests the results were not available: high level of lost samples and lost responses.
**Compliance to colonoscopy°**			
	49,1% (288)	(0,0 – 76,7)	Impossible to determine if missing data or low compliance.
Detection rate*			
Total	17,1/1000 (N 71)		Very few centres input data correctly and timely.
cancer	3,6/1000 (N 15)		
high grade adenomas	13,5/1000 (N 56)		
Low grade adenomas	N 17		
**Waiting time for colonoscopy§**			
within 30 days from FOBT	55,1%		Few colonoscopies due, but centres too busy. Very few centres input data correctly and timely.
31 and 60 days from FOBT	22,8%		
after 60 days from FOBT	22,1%		

° Based on 288 positive tests with complete follow up; high grade adenomas includes: 8% tubular with high grade dysplasia; 25% tubular-villous with high grade dysplasia; 24% tubular-villous with moderate dysplasia; 24% tubular-villous with low grade dysplasia; 4% villous with high grade dysplasia; 3% villous with moderate dysplasia; 1% villous with low grade dysplasia; 4% hyperplasic polyps; 5% 3 or more adenomas with low moderate dysplasia; 3% unknown. Low grade adenomas include: 73% of low grade and 23% of moderate tubular dysplasia; 4% unknown.

* Based on 4155 tests with full information.

§ Based on 140 colonoscopies with full information

$ Considering only the 6 centres that activated the collaboration with the GPs

Out of the 7196 samples returned, 288 could not be interpreted because the personal data or the sample itself was lost. Compliance to colonoscopy was very low, but during the site visits we observed that some colonoscopies had been performed without being registered; unfortunately it was impossible to quantify the phenomenon. Generally analyses of second level testing was limited due to the amount of missing information.

Carcinoma detection rates were 2.2/1000 and 2.4/1000 respectively for women and men, while the detection rates for high-grade adenomas were 11/1000 and 7.5/1000. Waiting times for colonoscopy varied greatly but tended to be long, with more than 22% waiting more than 2 months for colonoscopy.

The principal critical points noted during the site visits were: gastroenterology centre administrative staff related poorly to the healthy population, they had little experience managing the high number of patients and tests. Some of the centres were not easily accessible, i.e. hard to find, and required very short time for test pick up and return. There was such little time set aside for screening colonoscopies that the second diagnostic level was a bottleneck for the screening program, slowing down the rhythm of the entire screening process. Internet access at some of the centres was inadequate and unreliable, which prevented using the software.

## Discussion

### Choosing an organisational model: the grey areas

We thoroughly planned all stages of the screening process, taking into account its various aspects in the effort to establish the best baseline conditions for CRCS for our target population. To identify the best options we adopted the principles of evidence-based medicine, applying strategies that had preferably been tested in trials or, if trials were not available or not feasible, had solutions recommended by sound observational studies. We decided also to produce our own evidence if we noted contextual differences that hampered the generalizability of other studies' results (GP's effect on compliance and GPs' attitudes) [20] or if there was not enough literature on the topic (effect of the type of test on compliance, gastroenterologists' attitudes) [21]. Nevertheless, grey areas remain and some of the solutions were adopted arbitrarily.

The colonoscopy workload was identified as a critical point, consequently we needed almost all gastroenterology centres with medium-high volume of activity in our region to participate in the program independently of their administrative condition, in particular we were interested in involving university hospitals and clinical research hospitals, institutions usually not involved in breast or cervical cancer screening. With this aim we made three decisions: 1) we decided to put the FOBT reading machine (OC sensor) in the gastroenterology centre; consequently the system used 20 machines to interpret the test, an inefficient solution that increased the cost per test; furthermore gastroenterology centres do not have experience managing routine diagnostic tests and or handling a very high number of negative responses. 2) We decided to use the nearest gastroenterology centre as the test provider for the target population from non-participating GPs; other public clinics could be used to bring the test provider closer to the healthy target population. 3) As a consequence of the central roles played by the GP and the gastroenterology centres, the Local Health Unit had a marginal role in the screening program management.

Finally the economic incentive offered to the GPs was established with an agreement between the Regional Health Authority ant the GPs' trade unions, but we do not know if other forms of voluntary participation would be less effective.

### What did not work

First problem: only half of the centres chose to participate. One of the possible barriers that we identified is that the responsibility associated with prevention is unclear: in our health system the Local Health Unit is accountable for the health of the resident population within its borders, but most of the gastroenterology centres depends on the administrations of independent hospitals. In our model we tried to make the gastroenterology centre accountable for a preventive intervention and the LHU a sort of third player. This often resulted in a sort shifting of blame between the gastroenterology centres and the LHU.

Second problem: very few GPs agreed to participate, but wide variability was observed. It was clear that some LHUs did not have the administrative skills to enrol the GPs in the project, but all the coordinators working in the LHU screening programs (usually involved also in the organisation of breast and cervical cancer screening) reported that this task was difficult and time consuming.

We had great difficulty in determining how many people in the target population had been invited by the GP. This was the consequence of the freedoms obtained by the GPs' trade unions in the agreement: in fact, during the trial the target population that had their GP as test provider received a letter with a time schedule to pick up and return the FOBT; in the feasibility study we could not send mail to patients of the participating GPs because of union opposition. At the end of the round we were not sure if the GP's entire target population had had equal opportunity to be tested, generating an equality issue. The role of GPs in increasing screening attendance has been observed in several studies [26-30], but we faced so many difficulties that the advantages were largely outweighed by the problems; this is probably why other authors suggested the GPs' role be kept at signing the invitation letter [29,30].

Some LHUs had problems in respecting the 7 day time period to transport the faecal samples from the GPs' clinics to the gastroenterology centre.

The compliance in some gastroenterology centres was unacceptably low, confirming the results of the experimental phase [20,21]. Even though it was confirmed in the two settings of the experimental and pilot phases, the compliance rate obtained in our region by the gastroenterology centres is much lower than the Italian average (46%) [31]. These centres are used to treating a small number of ill patients and giving them a lot of attention, but they are not used to managing hundreds of people. Consequently they substantially reduced their availability to patients returning the FOBT.

The rhythm of mailing invitations to the target population had to be slowed in almost all centres because the colonoscopy workload was too high. This problem also occurred at centres that performed very few colonoscopies, revealing that they were not at all committed to screening and reserved very little time for this task. As a result, only one of the 20 centres invited the entire target population in the two year period.

Finally, there were also some problems with the management software. Some GPs did not enter all the required information, leading to the very confusing and unacceptable situation of sample results not being ascribed to a patient. Some gastroenterology centres had significant problems in entering colonoscopy results, mostly due to poor web connections, consequently the detection rates and colonoscopy compliance figures are not reliable. Nevertheless, the detection rate is in line with results of other Italian screening programs, while the compliance to colonoscopy is lower (41% vs 82%) [31].

## Conclusion (Lessons from failures)

By analysing all the problems we encountered, a single underlying cause is apparent: a lack of accountability. In our opinion, the program will work when responsibilities are clearly re-established, the most important and difficult being the institution's and staff's level of commitment to and involvement with the program. This is not the first experiment that has failed because of the difficulty in accountability of health services and their employees. The problem is more evident in the implementation of preventive interventions: in a CRCS program physicians do not have to face a health problem posed by the patient, but must themselves motivate prevention in the healthy.

We took a disease management approach [5] to ensure effectiveness, taking into account the role that the organisation of the health care system plays in determining quality and effectiveness [32,33]. Although the literature supports a disease-management approach, it does have some limitations. More general research is needed to assess effectiveness and the relative cost-effectiveness of different implementation strategies [34,35]; more agreement is also necessary on definitions and methodology [36].

## Conflict of interests

The authors declare that they have no competing interests, other than having contributed to the construction of the organisational model here analysed.

## Authors' contributions

AF, PGR, GG and PB conceived the experimental phase of colorectal cancer screening in Lazio and the pilot study. AF, PGR, AB and PB conceived the study and planned this analysis. AB, DB, SV and FQ conducted the pilot study and performed the data analyses. AF and PGR wrote the manuscript. The Colorectal Cancer Working Group conducted both the experimental phase and the pilot study at the peripheral level.

## Pre-publication history

The pre-publication history for this paper can be accessed here:


